# Behaviorist approaches to investigating memory and learning: A primer for synthetic biology and bioengineering

**DOI:** 10.1080/19420889.2021.2005863

**Published:** 2021-12-14

**Authors:** Charles I. Abramson, Michael Levin

**Affiliations:** a Department of Psychology, Laboratory of Comparative Psychology and Behavioral Biology at Oklahoma State University, United States of America; b Department of Biology, Allen Discovery Center at Tufts University, United States of America

**Keywords:** Behaviorism, synthetic morphology, biobot, memory, learning, basal cognition

## Abstract

The fields of developmental biology, biomedicine, and artificial life are being revolutionized by advances in synthetic morphology. The next phase of synthetic biology and bioengineering is resulting in the construction of novel organisms (biobots), which exhibit not only morphogenesis and physiology but functional behavior. It is now essential to begin to characterize the behavioral capacity of novel living constructs in terms of their ability to make decisions, form memories, learn from experience, and anticipate future stimuli. These synthetic organisms are highly diverse, and often do not resemble familiar model systems used in behavioral science. Thus, they represent an important context in which to begin to unify and standardize vocabulary and techniques across developmental biology, behavioral ecology, and neuroscience. To facilitate the study of behavior in novel living systems, we present a primer on techniques from the behaviorist tradition that can be used to probe the functions of any organism – natural, chimeric, or synthetic – regardless of the details of their construction or origin. These techniques provide a rich toolkit for advancing the fields of synthetic bioengineering, evolutionary developmental biology, basal cognition, exobiology, and robotics.

## Introduction

One of the most salient and interesting aspects of living things is their ability to learn from experience, exhibit preferences, and adaptively solve a diverse range of problems. The dynamic behavioral capacity of life forms is a central aspect of understanding evolutionary change and of efforts to control biological processes for beneficial applications in biomedicine and technology. The repertoire of behavior science is rapidly being expanded beyond the typical workhorse organisms (rats, etc.) to include a range of unconventional systems with rich behavioral capacities, including single cells, slime molds, plants, bio-hybrid robotics, and many others [1–18]. The sensors, effectors, and internal structures of these organisms may be quite different from those of typical animals studied by neuroscientists; thus, these systems present a challenge to the conventional approaches for characterizing intelligence and learning capacity, and to conceptual frameworks formed in the context of a fixed set of evolved, brainy creatures that have been produced in the biosphere to date [19–24].

**Figure 1.**
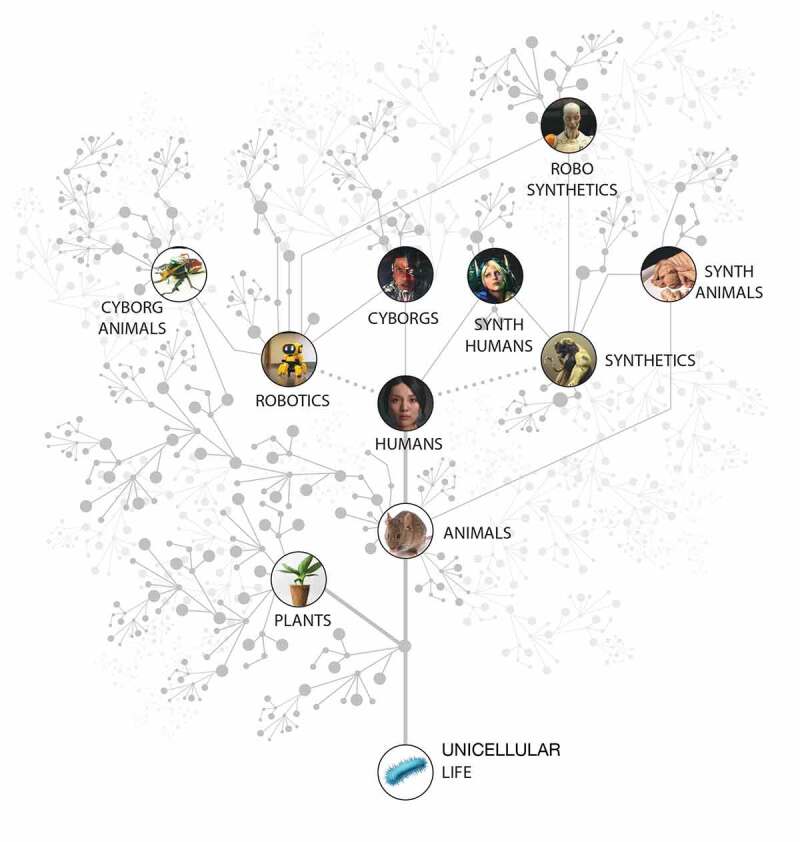
An expanded space of living systems as possible subjects of learning

**Figure 2.**
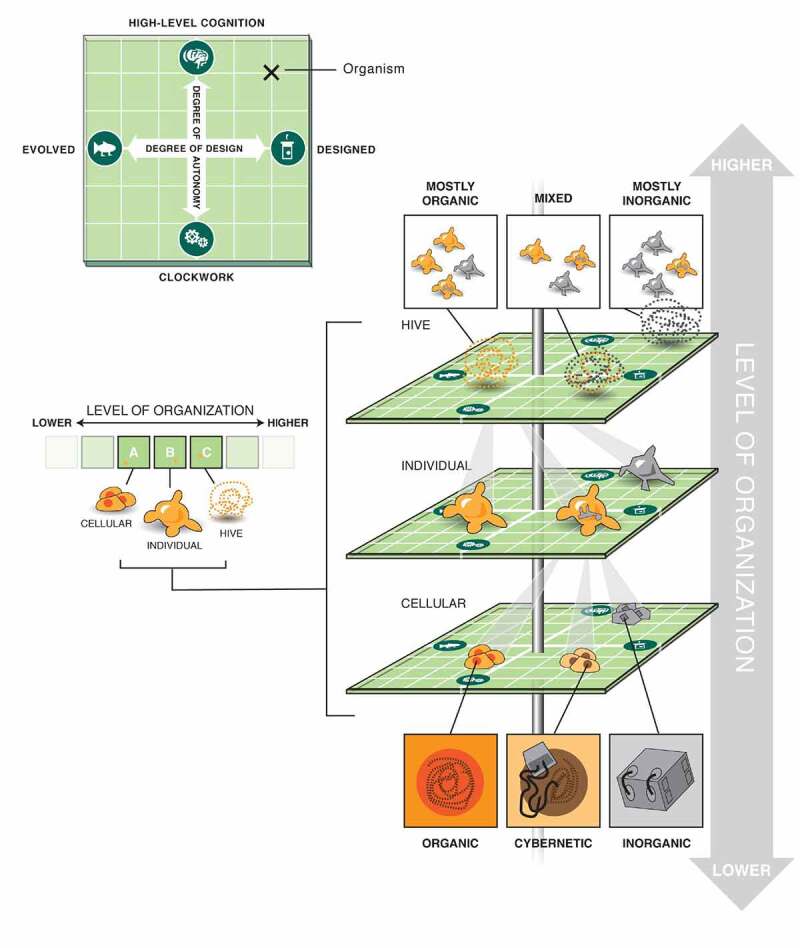
Multi-scale living forms

A plethora of novel biological systems are being produced by efforts in synthetic biology, artificial life, chimeric technology, and bioengineering [25–27] (Figure 1). Biobots, motile organoids, hybrots, cyborgs, chimeras, and other categories of living systems are now being made in laboratories, by combining organic cells and tissues from diverse species and incorporating inorganic components such as scaffolds, closed-loop software components, and electronic interfaces [28–30]. These organisms may contain various cell types (muscle, skin, etc.) and/or a range of smart materials and active matter [31–35], and each level of organization of such a system can be engineered, modified, or evolved independently. The demonstrated interoperability and plasticity of life gives rise to a huge option space of possible beings (Figure 2), which may be evolved, designed, or any combination thereof [32,36–41]. Remarkably, many such constructs are not merely passive tissues that implement self-assembly and physiology, but in fact exhibit various degrees of functionality such as motility, spontaneously-initiated behavior, and responsiveness to external stimuli. We refer to the whole class of possible active constructs, in whatever implementation, as “novel organisms” in our methodological discussion below.

In addition to the tree of life on Earth, bioengineering efforts are now producing chimeric living forms consisting of mixtures of evolved living material with designed components for novel sensor, effector, and computational capacity. This includes beings such as cybernetic organisms (cyborgs – animals with implanted technology that augments their capabilities), hybrots (brains driving vehicles or other physical systems instead of their default bodies [111–114]), and various synthetic creatures consisting of mixtures of genetic material from diverse sources which can also be rationally altered. The lines indicate relationships between the various categories, and the gray nodes schematize the near infinite variety of living beings that can be produced by recombination of evolved and designed subsystems. All of these will have their own problem space or Umwelt [115–117], sensory capacities, effectors, and functional IQs that span the gamut from very modest to highly advanced. Designing a framework for characterizing the learning capacities of such novel agents, where one cannot simply guess based on their resemblance to other standard forms or to a phylogenetic history, is essential. Figure courtesy of Jeremy Guay of Peregrine Creative.

The space of possible learning agents is astronomically large, because it is multi-dimensional: chimerism and hybridization with technology is possible on each level of organization of living systems. DNA, subcellular components, cells, tissues, organs, and whole organisms (alone or as part of hybrid swarms) – each level is itself a space of orthogonal possible options of evolved vs. designed, and passive vs. highly intelligent components. Choices made at one level can be combined with different choices of components at another level, providing an extremely vast option space for active systems that could exhibit learning (at any of its levels). Figure courtesy of Jeremy Guay of peregrine Creative.

**Figure 3.**
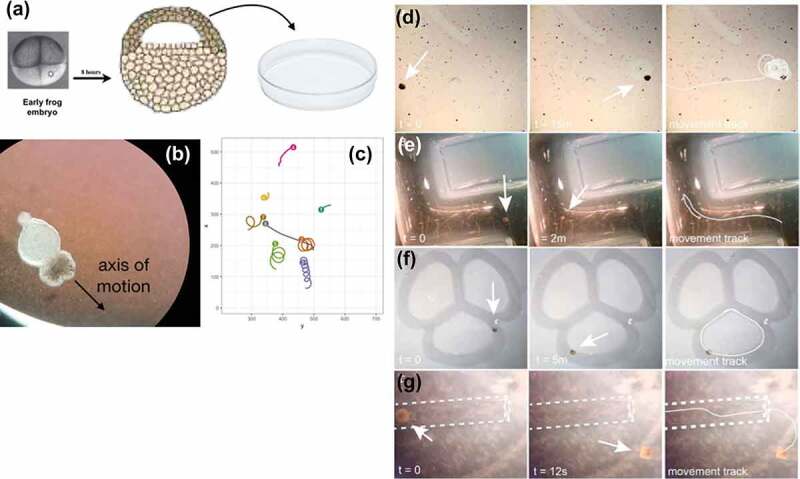
Xenobots, an example of a novel proto-organism with unknown learning capacity

**Figure 4.**
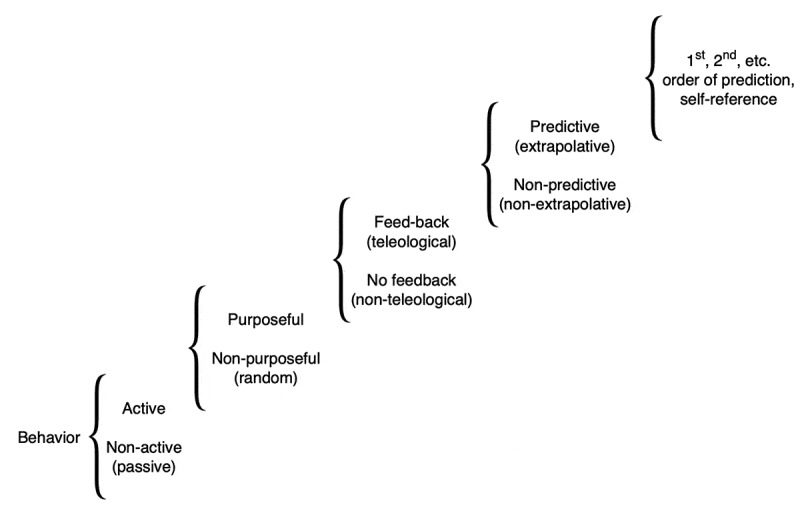
A scale of behavioral sophistication

For example, Xenobots [42,43] are self-propelled, autonomous proto-organisms made of epithelial and/or muscle cells that can navigate their environments and interact with each other in swarms, and perform actions that individuals could not do alone (Figure 3). It is imperative to begin to understand the degree and type of intelligence of such novel living beings, which are giving rise to an emerging interdisciplinary field at the intersection of cell biology, neuroscience, and engineering. How much sensing, decision-making, learning, and problem-solving do such systems exhibit? Developing a framework for empirically answering these questions, which could place any given new life form on a scale such as Rosenblueth et al.’s continuum (Figure 4) [44], is essential to progress in fields ranging from evolutionary ethology to soft robotics and machine learning.

Xenobots result when a frog embryo’s ectodermal cells are explanted into a petri dish (A), and sculpted in various ways as dictated by an evolutionary algorithm running in a virtual world [42,43]. They form a ciliated construct that swims on its own power (B) and exhibits all sorts of diverse motion patterns, especially when tracked in a swarm context (C, courtesy of Simon Garnier). Xenobots have interesting behaviors such as circling features in the environment (D, white arrow indicates a Xenobot orbiting a speck of material), traversing mazes (E, F), and moving through tubes (G). Their learning capacity is as yet unknown, and represents a key area for current investigation using the methods described herein. Panel A – from https://www.mussenhealth.us/growth-cones/conservation-of-neural-induction.html . Panels B,C courtesy of Douglas J. Blackiston, Levin lab. Panels D-G re-used with permission from [42].

Intelligent behavior is a continuum, which can be decomposed into major transitions (in the cybernetic tradition) ranging from purely passive behavior to responses to past stimuli that require diverse degrees of sophistication. Where a given novel system lands on this continuum cannot be determined by anatomical or phylogenetic data, but must be empirically determined by behavioral experiments. Figure after that in [44].

### What’s at stake: the impacts of an inclusive, general science of behavior

A number of disciplines will be strongly impacted by the developments of a rigorous science of behavior not tied to familiar organisms and brain structures, and freed from contingent assumptions about the material components essential for various degrees of functional sophistication.

The field of “basal cognition” [23,45–47] seeks to understand the phylogenetic origins of behavioral complexity, and is greatly enriched by the ability to make novel living beings in arbitrary configurations (for example, varying the amount or organization of neural components, or even producing entirely aneural systems) to more broadly probe structure-function relationships. Similarly, developmental neuroscience is concerned with the earliest stages of sensing and behavior during embryonic development, which shed light on functionality that is possible prior to the development of a complex brain. Soft robotics and Artificial Intelligence will greatly benefit from understanding engineering principles, inspired by emergent properties of novel life forms, that can be used to design and implement constructs with intelligent and problem-solving behavior. The characterization of degree of learning capacity is also of interest to exobiology (putative life found outside of Earth), as it may be a key criterion by which truly alien life forms could be recognized as such. Finally, learning capacity is fundamental to the ethics of organoids and synthetic biology, in terms of determining the degree of behavioral sophistication and thus framing our relationship to novel life forms, whether evolved, discovered, or engineered.

### Behaviorism: a useful tool for this new interdisciplinary field

A major roadblock to the characterization of learning capacity in novel constructs is that they often do not resemble any known model species used in neuroscience. Given a lack of precedent for existing training protocols within an astronomical option space of novel organisms, laboratories with expertise in bioengineering but not behavioral science often face a barrier for exploring the learning and behavioral capacity of new kinds of living organisms. Maximizing the positive impact of new bioengineering technologies requires a flexible, portable set of conceptual tools that focus on the essential functionality of learning, and that are not dependent on any assumptions about the structure or origin of the subject. Fortunately, there is an ideally appropriate formalism for this new field: behaviorism.

Behaviorist approaches are ideally suited to the new science of synthetic and chimeric organisms because they focus on observable functionality and are fundamentally agnostic about the internal construction of the subject, thus freeing researchers from brain-related assumptions that can constrain the study of novel creatures. Unlike the behaviorist tradition, cognitive approaches focus on inferring internal processes associated with information processing and are currently strongly associated with specific brain architectures, which many natural or bioengineered creatures will not possess [48–58]. Our goal here is not to review the large literature debating the relative merits of behaviorist vs. cognitivist traditions in neuroscience. Nor do we claim that this is the only approach to understanding novel living systems. Here, we offer another tool for the bioengineer’s toolbox, which facilitates focus on practical, functional analysis of capabilities of novel constructs.

The behaviorist approach avoids thorny philosophical issues of defining “cognition” in the context of sometimes minimal biological systems, or attempts to map their capacities onto familiar neural concepts, paradigms, and architectures developed for standard model species. We provide an overview of the conceptual and methodological tools that classical behaviorism has to offer the field of functional synthetic morphology, referring the reader to in-depth discussions of neglected aspects of invertebrate learning and the learning of plants [59–63] as precedents for even more profound extensions. We also discuss several methodological and conceptual issues that a bioengineer will face when designing learning experiments with novel organisms and provide practical strategies to help design a research program with novel organisms.

## Taxonomy of learning

One of the most important and interesting aspects of behavior is learning; thus, we begin with a taxonomy of concepts useful in the design of experiments to see how the behavior of a given living construct changes as a function of past experiences. Learning is classified as non-associative or associative. Nonassociative learning involves changes in the response to a single type of event, such as when the repeated presentation of a light alters the probability or strength of an orientation response to that light. It is considered the most basic learning process and serves as a building block for more complex learning. The two types of nonassociative learning that have received the most analyses are habituation and sensitization, discussed below.

Associative learning is a form of behavior modification involving the association of two or more events such as between two stimuli, a stimulus and response, or a chain of responses. In associative learning, the organism does learn to do something new or better. The three types of associative learning that have received the most attention are classical, instrumental, and operant conditioning.

Table 1 shows the type of conditioning and its relationship between nonassociative and associative learning. It is arranged from the simplest (habituation and sensitization) to the most complex (operant conditioning). When considering the table, it should be noted that each of the six conditioning categories can be made more or less complex. Consider the case of habituation. Here, an organism receives a repeated presentation of some stimulus until a behavioral response is no longer elicited. However, this situation can be made more complex if the experimenter simply adds context to the situation. For example, to design a habituation assay with “context”, one could perform the experiment in a chamber that contains some background stimulus such as a specific light intensity, temperature, and/or apparatus configuration (round vs. square). These specific background stimuli represent the context. When habituation is complete under the original context, a second context can be introduced (i.e., changes in temperature, light intensity, and/or apparatus configuration) and a comparison of habituation rates made between the two contexts. If the organism in question can process contextual information, habituation will be demonstrated in one context and re-learned in another.

**Table 1.** Types of learning

Conditioning	Association	Behavior	Examples
Habituation	Nonassociative	Nonarbitrary	Decrease in response
Sensitization	Nonassociative	Nonarbitrary	Increase in response
Alpha	Associative	US-US	Conditioned Sensitization
Classical	Associative	CS-US	Association of Stimuli
Instrumental	Associative	Nonarbitrary	BCC
Operant	Associative	Arbitrary	BCC

*Legend*: BCC = behavior controlled by its consequences. CS = Conditioned stimulus, US = Unconditioned stimulus

Not all animals show all types of learning, and it is advised to check new systems for all of them to survey its capabilities, starting with the simpler ones. In general, the more complex the organism the wider variety of learning it will exhibit. During an early survey phase, preliminary experiments can be performed without controls, to determine training parameters such as stimulus intensity and calibrate the assay. For experiments to be conclusive during the formal phase of the research process, appropriate control groups must be employed.

## Learning assays in novel organisms

Several fundamental learning paradigms can be used to study nonassociative and associative learning.

### Single subject or group designs

To carry out a behavioral experiment, one must make a decision early in the design phase whether the experimental design will employ a single subject design or a group design. In a typical single-subject design the subject serves as its own control. The single subject design has two benefits: it uses fewer organisms, and individual differences in a sample can more easily be controlled. If a single subject design is used, the organism receives two stimuli, one of which is followed by an event such as a reinforcement or by a US. In the case of classical conditioning, a CS followed by the US is known as CS+, and a CS not followed by the US is known as CS-. In the case of instrumental and operant conditioning, the stimuli are known as Sd and S-delta (S^Δ^), respectively. Statistical differences between the CS+ and CS- (or Sd and S^Δ^) serve as evidence for learning. The strongest evidence for demonstrating learning is obtained when the experimenter can employ both group and single subject designs.

### Instrumental and operant conditioning

A bioengineer considering employing situations in which the behavior of the synthetic organism is manipulated by the consequences of its actions must make a decision whether to employ instrumental or operant techniques. While instrumental and operant behavior are considered “behavior controlled by its consequences” the apparatus and research strategies are different. Apparatus associated with instrumental conditioning include such well known devices as the maze, runway, and shuttle box. In contrast, operant conditioning is most often studied in some version of the “Skinner box” (also known as an operant chamber). Unlike the apparatus used in instrumental conditioning, the Skinner box requires the experimenter to first train the organism to make some manipulative response such as pressing a lever. Only after the organism is trained to make such a manipulative response can the experiment proper begin. In general, how the experimenter trains the organism to press a lever (or make some other manipulative response) is not the primary interest of the experimenter. In contrast, all that is required to use the instrumental conditioning apparatus is that the designer organism be able to move from one place to another.

In addition to differences in apparatus, another difference between instrumental and operant behavior are the strategies used to examine learning. If a researcher is interested in how the organism learns, than the instrumental apparatus should be used. The reason for this is that the instrumental apparatus beaks down the behavior into parts or discrete units. Consider the case of a runway – a maze without choice points. The runway contains a start box, alley, and goal box segments. The behavior of the organism can be analyzed in each of the three segments in terms of such dependent variables as the time needed to leave the start box, the time required to transverse the alley and time required to consume the reinforcer in the goal box. In contrast, the Skinner box is best suited to measure changes in response rates. How the organism learns to press the lever is not the main interest. The main interest is how an independent variable influences the rate of a response – the dependent variable. The independent variable can either increase response rate, decrease response rate, or leave the response rate unchanged.

### Do instrumental and operant conditioning measure the same behavior?

A bioengineer reading a textbook on learning will generally find no distinction between instrumental and operant behavior as both are considered “behavior controlled by its consequences.” Indeed, the terms are often used interchangeably. We believe this is not correct. Although both instrumental and operant conditioning are behavior controlled by its consequences, the term operant behavior should be restricted to arbitrary behavior in which a manipulative response or skilled movement are used. On the assumption that developing a lever press or other manipulandum for a synthetic organism will be problematic, the question naturally arises whether the bioengineer can create a situation where an instrumental response is arbitrary.

An instrumental response can be shown to be arbitrary if some property of the response can be manipulated. For example, if a novel organism can be shown to increase or decrease its speed of movement as a result of the contingency of reinforcement or learn a series of correct turns in a complex maze, this would turn movement into an arbitrary behavior. The key phrase to help the bioengineer to distinguish instrumental and operant behavior is whether the organism in any given apparatus “can show you it knows how to use it.”

A good illustration of the distinction between instrumental and operant behavior is the “hunt and peck” method of typing on a keyboard. The reinforcement is typing the correct letter – an instrumental response. However, with training, the “hunt and peck” method is replaced with a series of rapid and coordinated movements – an operant response. If a synthetic organism cannot make a manipulative response, there are a series of procedures that use the runway to study operant schedules of reinforcement. For example, rather than pressing a lever, an organism might receive reinforcement on the 5^th^ run (i.e., trial) through the runway. This is known as a “fixed ratio – 5 schedule of reinforcement [64].

### Novel sensory-motor paradigms

How can the experimenter explore learning when one does not know in advance what the animal perceives of the outside world, or which stimuli will have salience to a creature that does not have eons of evolutionary pressure behind it for specific behaviors? Many of the synthetic living organisms have unusual sensory or effector capabilities. They may have natural or artificial (bioengineered) sensors of magnetic fields, light in unusual wavelengths, vibration, novel chemical receptors, or may be instrumentized by electrodes to respond to stimuli that exist in a virtual world (for example, like sensory substitution [65–68], but the stimuli could come from unconventional spaces such as stock market data or physiological parameters of another life form). Their behavioral output may be muscle activity, or it may be electric signals that are amplified and used to drive a vehicle, control some other animal’s habitat, or run a 3D printer to modify the environment.

Picking appropriate stimuli and testing paradigms for entirely novel organisms is largely a matter of trial and exploration. The stimuli selected during the initial pilot experiments will be based on knowledge of its component cells and tissues, but it can be very hard to extrapolate from that to system-level behavioral capacities. As the study of animal learning has a long and rich tradition [69], we strongly recommend that the researcher collaborate with a comparative psychologist at least in the initial phase of the experiments [70] as analogies might be useful to unconventional models such as various invertebrates, plants, etc.

When working with novel organisms, the researcher must be aware that the organism may be less sensitive to environmental contingencies. Such decreased sensitivity may, for example, result in an inability to associate conditioned and unconditioned stimuli in a classical conditioning situation or a response and reinforcer in an instrumental or operant situation. Such results may be deceptive. In a lever press situation developed for crabs, it was found that restraining the crab with clamps produced poor results but enclosing them in a small box produced effective lever pressing [71]. As much as possible, organisms should be allowed to interact with the world and choose which signals are salient.

A similar situation was found with the proboscis conditioning of stingless bees. Restraining stingless bees in tubes did not produce any proboscis conditioning but putting them in small bottles where they made contact with the stimuli through a screen produced rapid learning [72]. We recommend that a catalog of stimuli, responses, and training situations be created for synthetic organisms and that this catalog be shared with the scientific community.

In our view, creating such a “behavioral catalog” is possibly one of the more exciting aspects of the work with new organisms. The researcher should try a variety of stimuli such as light, magnetic field, and vibration. The stimuli initially selected will be based upon the design of the organism. Each stimulus should be tested systematically at a range of intensities similar to that used in psychophysics experiments. Moreover, we recommend that detailed behavioral records be kept describing the organism’s reactions to the stimuli. The researcher, thus furnished with an empirically based data-set complete with detailed observations, will then be sufficiently informed to design non-associative and associative learning experiments.

### Habituation and sensitization

We recommend beginning with habituation experiments (i.e., nonassociative learning). The rationale for this is fivefold. First, only one stimulus is needed. The use of only one stimulus, repeatedly presented, reduces the complexity of the experiment. Second, nonassociative learning shares many properties of associative learning including spontaneous recovery, generalization, and stimulus intensity effects [73]. Third, the habituation experiment can guide the researcher in the selection of training variables for associative learning experiments. For example, if it takes the synthetic organism 40 trials to reach some habituation criteria to light, then the researcher knows that light can be used as an unconditioned stimulus for at least 30 trials (assuming that the criterion for habituation is ten consecutive no responses). It makes no sense to design a classical conditioning experiment until the researcher knowns how effective the unconditioned stimulus is. These data can be obtained in a habituation experiment. Fourth, habituation has been studied in both invertebrates and vertebrates for decades and there are literally hundreds of published experiments to which the results from the new organism can be compared. Fifth, the habituation paradigm can be converted to an associative learning paradigm by the addition of context. When the synthetic organism demonstrates habituation in one context, such as color, temperature, or shape of apparatus, and is then placed in a second context, is habituation maintained or does the organism need to re-learn to habituate in the new context? We discuss habituation in context below.

The other widely studied non-associative learning paradigm is sensitization. Sensitization experiments also require the presentation of only a single stimulus. The results of repeatedly presenting the stimulus is that a reaction will develop as the number of stimulus presentations is increased. Like habituation, sensitization also has advantages for the design of associative learning experiments. One principal advantage is the design of alpha conditioning experiments also known as conditioned sensitization. As discussed previously, here, instead of associating a neutral stimulus (CS) with a non-neutral stimulus (US), two US’s are associated. The two US’s can be from the same sensory modality (example, a low intensity vibration followed by a strong intensity vibration) or a different modality (example, light and vibration). The intensity of the first is lower than the intensity of the second. After a number of pairings, the first stimulus should evoke a reaction similar to the second stimulus. As long as appropriate controls are used, alpha conditioning is an example of associative learning. Alpha conditioning is especially important if a novel organism does not respond to neutral events.

## How do you motivate a novel organism?

Related to the question “How do you explore learning when you do not know in advance what the animal can perceive?” is the key question of how to motivate a new type of organism. A rich literature addresses the conceptual issues around preferences and valence in minimal living [74] and even non-living (e.g., AI) [75,76] systems; here, given the focus on novel biological constructs, we use a functional definition of motivation with respect to substances, states, or signals that are necessary or contrary to the system’s longevity and well-being. An organism’s motivational state is manipulated by depriving it of some commodity, using a preferred commodity, or by varying the intensity of an aversive event. If a behavioral catalog is created and shared, the answer(s) will readily be revealed. The question of motivation is especially important for instrumental and operant experiments because behavior in these two paradigms is controlled by its consequence. The consequences will be the contingent application of either appetitive stimuli (such as food) or aversive stimuli (such as electric shock).

### Appetitive stimuli

One way to search for appetitive stimuli is to determine what the organism “covets.” This can be done in the course of constructing a behavioral catalog. For the vast majority of learning experiments, food is the appetitive stimulus of choice. The use of food has several difficulties, including satiation effects and the need to use apparatus to deliver the food. For food to be effective, the organism must be food-deprived. Even synthetic organisms will have metabolic limitations, and often can be deprived of nutritional resources. An alternative to food deprivation is to use a preferred food. One interesting challenge of working with synthetic organisms is the search for unique and novel appetitive stimuli. Until such stimuli are found, we recommend the use of aversive stimuli.

### Aversive stimuli

Motivation is generally not an issue with habituation, sensitization, alpha conditioning and classical conditioning experiments. The motivation in these experiments comes from the termination of the unconditioned stimuli themselves. Motivation is important in instrumental and operant experiments; as these experiments are based on “the control of behavior by its consequences”, the consequences have to be “coveted.”

The use of aversive stimuli has much to recommend it at the initial stages of experimentation, and can be more effective than rewards. An aversive stimulus such as electric shock is easy to administer and control. It can be precisely turned on and off without after-effects such as those associated with changes in temperature. Shock can also be easily incorporated into instrumental and operant conditioning experimental designs associated with escape, punishment, and avoidance. One decision the researcher must make is whether to use alternating current (AC) or direct current (DC) shock. We recommend DC current as it is readily produced by batteries and therefore easier to use than AC current. Direct current is also less dangerous to use than AC. Whether AC or DC current is used, the researcher should run pilot experiments to determine the minimum amount of shock that can be used to elicit a response from the organism.

### Escape

Of all the available instrumental and operant conditioning procedures, we recommend the study of escape during the pilot phase of experimentation. Escape can be considered as an example of appetitive conditioning, with the difference being that the reinforcement is the termination of the aversive event rather than the receipt of an appetitive stimulus. In this case, the appetitive reinforcement is “shock free time.” The escape paradigm can be used to investigate many phenomena of appetitive conditioning including the delay between the response and the cessation of shock, reinforcement schedules, reinforcement magnitude, and rate of reinforcement.

### Punishment

Punishment is another associative learning paradigm that can easily be studied with synthetic organisms. In punishment, a specific response (such as crawling) produces the delivery of an aversive event. If punishment is effective, the organism will stop making the response relative to unpaired controls. As in the escape paradigm, punishment can be used to study the delay between the response and the cessation of shock, reinforcement schedules, reinforcement magnitude, and rate of reinforcement. Perhaps the easiest punishment paradigm to use is to first determined if the synthetic organism is unconditionally attracted to some stimulus such as light. When the organism moves toward the light, it is punished with a shock. After a number of light-shock pairings, the organism should stop moving toward the light. If a group is included that receives unpaired light and shock (these animals should continue to move toward the light) and a statistical difference is revealed between the two groups, punishment is demonstrated. Motivation can easily be studied in this paradigm by just varying the light intensity. A pilot experiment should first be run to determine the speed in which the designer organism is attracted to light of various intensities (or some other stimulus the organism is attracted to). Motivation can then be studied as a function of the light intensity.

### Avoidance

The most common example of avoidance conditioning is known as signaled avoidance. In the signaled avoidance paradigm, a cue or CS is presented and if the organism responds to the signal the aversive event is omitted or postponed. It is important to note that until the organism begins to respond to the signal, the first few trials resemble the classical conditioning paradigm (the CS is paired with the shock). However, if a response is made to the CS the response avoids or postpones the aversive event. The avoidance paradigm is an interesting blend of classical and instrumental conditioning. It resembles classical conditioning in that a CS and US are presented, yet it contains an instrumental component in that a response to the CS avoids or postpones the US.

### General activity conditioning

It often goes unrecognized that until the organism responds to the CS, the avoidance paradigm is a straightforward application of classical conditioning. General activity conditioning is one of the simplest of the classical conditioning paradigms. In this paradigm a stimulus (CS) is paired with a brief electrical shock (US). The amount of general reactivity to the CS is measured on each trial. If conditioning is evident, the organism will increase its reactivity to the CS until some asymptote is reached compared to unpaired or discrimination control groups. To modify this paradigm for avoidance learning all that is needed is a contingency where, if the organism responds to the CS, the aversive event avoided or postponed.

## Designing Pavlovian and Instrumental/operant experiments

This section provides an overview of some factors that must be considered in the design of an initial demonstration of Pavlovian and operant/instrumental experiments. Additional details on experimental design can be found in [59,71,77]. A low cost automated system for programming conditioning experiments is available [78,79]. As apparatus for many species have not yet been developed, we recommend using 3d printing [80]. We also recommend object orientated modeling (OOM) for analyzing the data [81,82]. OOM has the advantage that no statistical assumptions are necessary and P values are not used.

- Pavlovian conditioning (Forward conditioning)
  1. Select conditioning protocol [83]
  2. Find a CS that is neutral. If none can be found use an alpha conditioning procedure (US-US conditioning)
  3. Find a US that provides a consistent and reliable unconditioned response.
  4. Choose CS and US intensity, intertrial interval (ITI) and interstimulus interval (ISI). The ITI (time from the end of the US to the beginning of the next CS) should be relatively long to avoid effector fatigue and sensory adaptation. In contrast the ISI (time from the end of the CS to the next US) should as short as possible.
  5. Select whether you will measure conditioning on each trial (trial by trial method) or on a select number of trials (test trial method). We recommend the trial by trial method.
  6. Select whether the experiment will end after a fixed number of trials or if some conditioning criteria is met (ex. 10 consecutive conditioned responses).
  7. Determine if an extinction procedure will be used following acquisition training. There are two methods of extinction: the most common is to omit the US (CS only), and the other is to unpair the CS and US (unpaired method). The unpaired method of extinction is seldom used. It is important to include an extinction component for two reasons. First, the extinction data will provide important information on the persistence of the CR (conditioned response), and second, it will provide data for future experiments on inhibitory conditioning.
  8. Incorporate control groups. We strongly recommend that during the initial demonstration multiple control groups be used. These control groups are CS only (provides information whether the organism’s response to the CS increases over the course of the experiment and sets the stage for possible experiments on latent inhibition), US only (provides information that the unconditioned response remains robust and stable throughout the experiment and sets the stage for possible experiments on the US pre-exposure effect) and “blank.” The blank group serves as an activity control. In addition to the CS only, US only, and blank groups, unpaired and discrimination groups should also be included. If using a group design, the unpaired group is crucial as it contains both the CS and US and can be directly compared to the CS-US paired group.

When using the unpaired group, a decision must be made as to the order of presentations of the unpaired stimuli. We recommend sequences of “ABBA BAAB” where A is the CS and B is the US. If additional trials are needed the sequence is repeated. It is important to note that the unpaired group will have twice as many trials as the paired group because, in contrast to paired animals, the CS and US are presented individually on each trial. For example, on trial 1 the CS is presented and on trial 2, the US is presented (CS, US, US, CS, US, CS, CS, US).

When designing the unpaired control group, a decision must be made as to the time between the CS and US. We recommend that the time between stimuli be half the ITI of the paired group. This will ensure that the time between CS presentations closely approximates the ITI used for the paired group. For example, if the ITI is 10 minutes for the paired animals, it will be 5 minutes for the unpaired animals. An additional rationale for using an unpaired control group is that, if this group subsequently receives paired training, conditioned inhibition can be studied. While not typically used during an initial demonstration of Pavlovian conditioning, we recommend using a discrimination group. In this case two CSs are used. The CS paired with a US is called CS+ and a CS not paired with a US is called the CS-. The ABBA BAAB pseudorandom sequence can also be used to present the CS+ and CS- (CS+, CS-, CS-, CS+, CS-, CS+, CS+, CS-). Once again, the ITI should be half that used for the paired animals. The discrimination group provides all of the advantages offered by a single subject design (i.e., each subject serves as its own control). Moreover, if the CS+ and CS- are reversed after initial training (the CS+ now becomes CS- and CS+ now becomes CS-) reversal learning can be studied. Differences between paired and unpaired groups and between CS+ and CS- provide the strongest evidence of conditioning.

After the initial demonstration of classical conditioning as recommended here, the researcher can then explore other conditioning protocols. Some of the more interesting include:

*a. Trace conditioning*. In trace conditioning the CS terminates before the onset of the US. The gap between the end of the CS and onset of the US is called the trace interval. This procedure can be used to study memory. The non-overlap procedure can be considered as trace conditioning with the time between the end of the CS and onset of the US as “0”.

b. *Temporal conditioning*. In temporal conditioning there is no explicit CS. Rather, the US is presented at regular intervals and the presence of a conditioned response is noted as the presentation of the US approaches. This procedure is useful to determine whether the organism shows timing behavior.

(B) Instrumental/operant conditioning
  1. Determine if you are studying instrumental or operant conditioning. If the response is arbitrary, standard control groups are not necessary as no animal will make the required response without specific training. In effect, an animal that can make an arbitrary response is serving as its own control.
  2. Select reinforcers and discriminative stimuli. If no positive reinforcers can be found, escape conditioning can be substituted. In escape conditioning, the reinforcer is time away from the aversive event. The discriminative stimuli (Sd) will serve as a cue to set the occasion for the response. The use of discriminative stimuli is not required during an initial demonstration but if responding can be restricted to the presence of the Sd, this provides strong evidence of conditioning.
  3. Select the training apparatus. There are several choices for apparatus including shuttle boxes, runways, mazes, and operant chambers. Many of these apparatus can be 3d-printed.
  4. Incorporate control groups. The control groups will be like those used in Pavlovian conditioning assuming that an arbitrary response cannot be found. We would recommend: 1) response only (no consequence) and 2) reinforcement only. In addition, there must be a control group that receives an unpaired presentation of response and reinforcer.

### The unpaired control group

**Figure 5.**
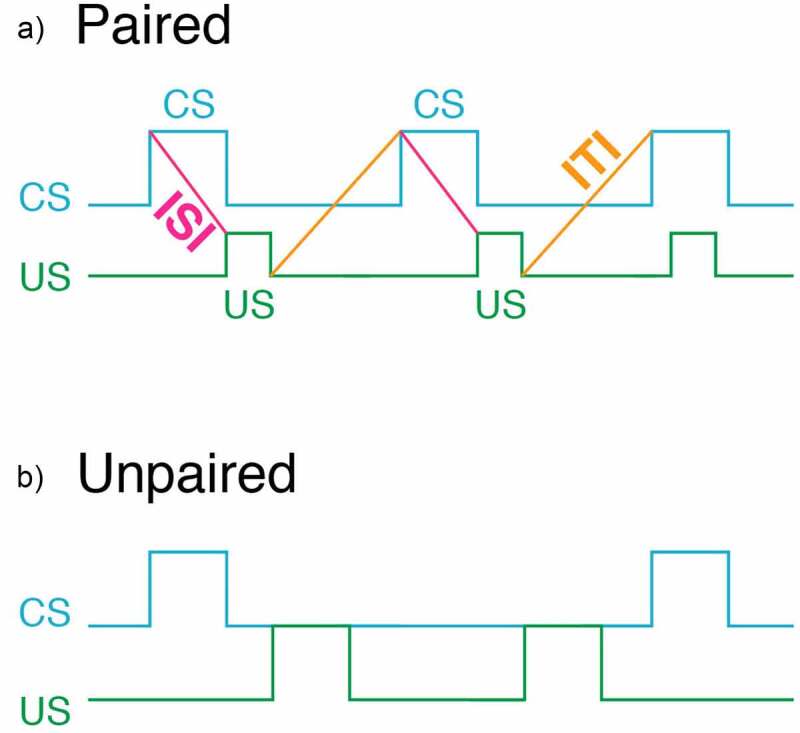
A schematic diagram of the sequence of the CS and US in both paired (a) and unpaired (b) training. We advocate using the “non-overlap” procedure. In this procedure the CS terminates prior to the administration of the US. This procedure has the advantage that conditioned responses are easily observed without the presence of the US. In the “overlap” procedure the US is presented sometime during the presentation of the US

When determining whether a novel organism exhibits associative learning, it is best to employ an unpaired control group. For example, if a researcher is interested in demonstrating classical conditioning (also known as Pavlovian conditioning), the control group should receive unpaired presentations of the conditioned (CS) and unconditioned stimulus (US) (Figure 5). The pairing of the CS and US is known as a training trial and the time between the training trials is known as the intertrial interval (ITI). When using the unpaired control group, the ITI should be half of that used for a paired training group.

The rationale for using an ITI that is half that used in the paired group is to keep the time between CS presentations roughly equal. If the control group uses an ITI that is the same as that used for the paired group, the control group will have an ITI that is twice as long as that used for the paired group. This is because the paired group received both the CS and US on each trial (i.e., they are paired). In contrast, only a CS or a US is presented during a trial (i.e., they are unpaired). For example, if a researcher has 10 training trials for the paired group, there will be 20 trials in the unpaired group (10 CS trials and 10 US trials).

The use of the unpaired control is also necessary to determine whether alpha conditioning is learned. Alpha conditioning is an example of conditioned (i.e., learned) sensitization and is seldom studied in the contemporary learning literature. We feel this is unfortunate because when working with novel organisms, the researcher may find that the organism does not respond to neutral stimuli. Classical conditioning involves pairing a previously neutral stimulus with a response producing stimulus [55]. In alpha conditioning, the association is between two non-neutral stimuli. In this case, “non-neutral stimuli” refers to stimuli that elicit a response resembling a conditioned response (i.e., a learned response) without the benefit of training. Consider the case where two different light intensities are paired and both intensities elicit eye closure albeit one elicits a slight twitch and the other a full closure. The first presentation of the low intensity stimulus (US_1_) elicits a response resembling the conditioned response (CR) without the benefit of training. Following the presentation of the low intensity light, a high intensity light is presented (US_2_). This high intensity light should elicit a vigorous response. After a number of US_1_-US_2_ pairings, the organism elicits a vigorous response to US_1_. Whether this is learned is demonstrated with the unpaired control group.

The logic of the unpaired control group is also used in instrumental runway experiments. A reinforcement is placed at the end of the runway and over successive runs, the organism speeds up to some asymptote. Without an unpaired control group that receives reinforcement in different parts of the runway, such as in a “goal box” on some trials and in an alley on others, it cannot be unequivocally concluded that reinforcement in the goal box produces the increased running speed. The increase in running speed can easily be interpreted as simple escape behavior. However, the increase in running speed would be an example of learning if the running speed of the unpaired control group is significantly less than the experimental group.

## Future developments of behaviorist tools for synthetic bioengineering

The ability to construct an endless variety of novel “model systems” provides a rich opportunity to improve the state of the art in behavioral science.

### Taxonomies of learning paradigms need to be developed

The confusion related to the various definitions of conditioning is problematic for a more inclusive science of behavior. This confusion is becoming more pronounced as behavioral neuroscientists interested in learning begin to enter the field. As the current zeitgeist is to interpret all behavior in terms of cognitive processes, they are not exposed to the alternatives provided by behaviorist interpretations [49]. We believe that one way to solve this problem is to do away with behaviorist and cognitive interpretations and focus on the description of paradigms used to generate the behavior in which the researcher is interested (i.e., behavioral taxonomies). As Bitterman noted almost 50 years ago, “Classification is not merely a matter of taste” [84] (pg. 81). We believe that many of the problems associated with definitions of conditioning phenomena can be avoided if researcher link a conditioning procedure to a behavioral taxonomy. Several such have been proposed [83–86] but none are in consensus use. The addition of novel, engineered life forms to this field is sure to trigger additional discussion aimed at defining truly inclusive and general taxonomic frameworks for wide-ranging types of behavior.

### The need to report individual level data

The “operant conditioning journal” known as the *Journal of the Experimental Analysis of Behavior* historically contains many examples of the individual performance of various vertebrates including monkeys, pigeons and rats, which can be used as inspiration for designing novel experimental designs. The book *Schedules of Reinforcement* [87] contains hundreds of examples of individual performance of pigeons in the form of cumulative records (a curve showing the number of responses across time). This book is also unique in that not only is the focus on individual performance but the book describes many types of schedules of reinforcement that go far beyond the simple schedules of reinforcement described in this paper. For example, in a conjunctive schedule, a reinforcement is delivered after the requirements of both a ratio and interval schedule of reinforcement are satisfied. *Schedules of Reinforcement* is an excellent source of ideas and protocols for the study of operant conditioning in synthetic organisms and provides the researcher with many graphic examples of the importance of including examples of individual data.

In contrast to vertebrate studies of learning – especially in operant conditioning – there are few reports containing individual data in invertebrate models. Many invertebrate learning experiments present data in the form of group curves. Such aggregate data provides little information regarding an individual’s performance or variation among animals. Moreover, in some cases the data of individual animals that appear to be outliers are discarded. We view this as inappropriate because much information about functional heterogeneity, the role of noise, and the relationship between genetics and behavior can be obtained from individuals considered outliers.

With some exceptions there are few examples of individual data for the *Limax, Aplysia, Apis*, and *Hermissenda* learning models [60]. Examples of individual data are available for the classical conditioning of proboscis conditioning in honey bees and operant conditioning in both crabs and honey bees [71,88,89]. We recommend that researchers studying the learning of novel organisms publish examples of individual data and upload all of the data as a Supplement to enable others to mine it in new ways.

### Definitional and taxonomic issues

Table 1 is a bit misleading in that it presents the various conditioning paradigms as unequivocally distinct examples. In fact, there are no consistent, universally agreed-upon definitions of conditioning phenomena. The problems associated with inconsistent definitions have stimulated debate related to, for example, the definitions of species, sexual selection, eusociality, and tool use [90].

Surprisingly, among contemporary behavioral scientists, with few exceptions, there has been little debate or recognition of the inconsistent definitions of conditioning procedures. One of the most egregious examples is classical conditioning. Researchers working with novel organisms must understand that contemporary accounts of classical conditioning often fail to mention that there are at least four different methods to produce classical conditioning. These methods can be distinguished based on the degree of experimental control and the relationship between the conditioned and unconditioned response [83]. There is no research directly comparing these procedures and it is doubtful that these four methods all produce the same behavioral phenomena – i.e., classical conditioning. The conditioning methods range from general activity conditioning, suppression of ongoing behavior, autoshaping (learning an operant response through classical conditioning), and situations where the unconditioned stimulus is directly injected into the organism.

Another definitional issue concerns operant conditioning. For many researchers, operant conditioning is any “behavior controlled by its consequences.” Seldom discussed, the hallmark of operant conditioning is whether the organism can not only demonstrate the use of a manipulandum such as a lever, but that it can also use that manipulandum in novel ways. Novel behaviors can be demonstrated by training the organism to manipulate a device with different degrees of force, moving the device up or down or from side to side. Such behavior is easily observed in most vertebrates but has never been demonstrated in an invertebrate.

Examples of “operant” conditioning in invertebrates include using punishment to manipulate eye position in a crab, negotiating a maze, running down an alley, and running against a preference [59]. It is often forgotten that the rationale for using a lever or some other manipulandum is that species-typical behavior is minimized. If a manipulandum cannot be used, there is set of runway procedures that are analogous to the operant methods [64]. For example, if a researcher is interested in studying a situation where a reinforcement becomes available after a specified number of responses since the previous reinforcement (known as a Fixed Ratio schedule of reinforcement), all that needs to be done is to present the reinforcement after the required number of trips through the runway. Even here, care must be taken to ensure that what is being manipulated is operant behavior rather than a simple instrumental response.

While it has been repeatedly demonstrated that an invertebrate can increase its running speed to some asymptote in the pursuit of food [59], it has not been shown that an invertebrate can adjust its speed to procure the food. It is this adjustment in response to the contingencies of reinforcement which transforms the instrumental behavior of running down the alley into an operant behavior.

Consider the head turning response in *Aplysia* (sea slug). If turning its head to the right is punished, the *Aplysia* will quickly learn to keep its head to the left. To truly make it an example of operant behavior, what would be needed is a demonstration that the *Aplysia* can vary some aspect of its head turning response such as speed or duration. In situations such as these we refer to the behavior as instrumental conditioning.

## Discussion

Learning capacity, in the sense of specific changes of future responses in light of past experiences [91], has been suggested in many unconventional substrates, including cells [92–96] and even subcellular components such as molecular pathways and gene regulatory networks [97–102]. Moreover, synthetic biology is increasingly providing micro-designed [37,38,103–106] or emergent [42,43] active living constructs. The space of possible subjects for learning experiments is vast, and is growing all the time given developments in smart materials, synthetic bioengineering, brain-computer interfaces, and other fields. The ability to train synthetic living machines for useful functions [25–27] will be a very important new toolbox for the bioengineer, in addition to the design of novel bodies with hardwired operation. It is also likely to have implications for machine learning and robotics, as novel learning architectures discovered in aneural and neural systems could improve performance if imported to silicon-based (or other) media.

Developing effective training protocols for novel model systems is important in two other broad ways. First, it will shed essential light on the fundamental aspects of learning, distinct from the frozen evolutionary accidents of the phylogenetic history of life on Earth. Indeed, many debates about the locus and mechanism of memory could be enlightened by experiments in unconventional substrates [107–109]. Second, training offers workers in regenerative medicine and bioengineering a path toward outcomes that are too complex to micromanage by physical (genetic, pathway) rewiring. By exploiting the learning and basal problem-solving capacities of cells and tissues (in vivo or in vitro), biomedical strategies could push much of the complexity onto the system itself: using stimuli, not hardware rewiring, to achieve desired endpoints such as specific morphogenetic outcomes, whether in the patient or in synthetic living machines with useful functions [110]. Much as evolution exploits learning to achieve outcomes far faster than is possible at the genetic level alone, scientists and engineers can leverage the same advantages to overcome the inherent complexity of the mapping between biological structure and function.
